# Using the RE-AIM framework to evaluate the feasibility of a parent-focused intervention targeting childhood obesity

**DOI:** 10.1186/s40814-023-01248-8

**Published:** 2023-03-13

**Authors:** Daniel Briatico, Kristen C. Reilly, Patricia Tucker, Jennifer D. Irwin, Andrew M. Johnson, Erin S. Pearson, Dirk E. Bock, Shauna M. Burke

**Affiliations:** 1 Faculty of Health Sciences, Western University, London, Ontario Canada; 2 Children’s Health Research Institute, Lawson Health Research Institute, London, Canada; 3 Faculty of Health and Behavioural Sciences, Lakehead University, Thunder Bay, Ontario Canada; 4 Department of Paediatrics, Schulich School of Medicine & Dentistry, Western University, London, Ontario Canada; 5 Office for Child and Adolescent Medicine, Windisch, Aargau Switzerland

**Keywords:** Childhood obesity, Intervention, Community, Parent-focused, Program evaluation, RE-AIM, Health-related quality of life, Feasibility

## Abstract

**Background:**

Childhood obesity remains a serious public health concern. Community-based childhood obesity treatment interventions have the potential to improve health behaviors and outcomes among children, but require thorough evaluation to facilitate translation of research into practice. The purpose of the current study was to determine the feasibility of a community-based, parent-focused childhood obesity intervention (“C.H.A.M.P. Families”) using the RE-AIM framework, an evaluation tool for health interventions.

**Methods:**

A single-group, non-randomized, repeated measures feasibility study was conducted. Participants (*n* = 16 parents/caregivers of 11 children with obesity) completed a 13-week parent-focused education intervention. The intervention consisted of three main components: (a) eight group-based (parent-only) education sessions; (b) eight home-based (family-centered) activities; and (c) two group-based follow-up support sessions for parents and children. The five dimensions of RE-AIM—*reach*, *effectiveness*, *adoption*, *implementation*, and *maintenance*—were assessed using various measures and data sources (e.g., child, parent/caregiver, costing, census) obtained throughout the study period. Outcome variables were measured at baseline, mid-intervention, post-intervention, and at a 6-month follow-up.

**Results:**

Overall, the C.H.A.M.P. Families intervention reached approximately 0.09% of eligible families in London, Ontario. Despite the small number, participants were generally representative of the population from which they were drawn, and program participation rates were high (*reach*). Findings also suggest that involvement in the program was associated with improved health-related quality of life among children (*effectiveness*/*individual-level maintenance*). In addition, the intervention had high fidelity to protocol, attendance rates, and cost-effectiveness (*implementation*). Lastly, important community partnerships were established and maintained (*adoption/setting-level maintenance*).

**Conclusions:**

Based on a detailed and comprehensive RE-AIM evaluation, the C.H.A.M.P. Families intervention appears to be a promising parent-focused approach to the treatment of childhood obesity.

**Trial registration:**

ISRCTN Registry, Study ID ISRCTN 10752416. Registered 24 April 2018.

## Key messages regarding feasibility

- What uncertainties existed regarding the feasibility?

While there is evidence available in support of parent-focused childhood obesity treatment interventions, information related to program implementation and sustainability in community-based settings is lacking and/or underreported in the current literature.

- What are the key feasibility findings?

The findings outlined in this study provide preliminary support for the feasibility of C.H.A.M.P. Families and suggest that this parent-focused, community-based childhood obesity program could lead to improvements in children’s health-related quality of life.

- What are the implications of the feasibility findings for the design of the main study?

Via a comprehensive examination of RE-AIM dimensions, the feasibility findings presented in this manuscript can be used to inform the development and implementation of future community-based pediatric obesity interventions. We also expect that these findings will inform the planning and design of a future RCT conducted with a larger and more diverse group of children and families.

## Background

Obesity is widely recognized as one of the most significant health problems affecting children in the twenty-first century [1]. In recent decades, the prevalence of overweight and obesity in children has increased dramatically, affecting approximately 340 million children worldwide [1]. Accompanying the prevalence of obesity among children is the associated health risks including a myriad of physical [2–4] and psychological health sequelae [4–6], as well as considerable economic implications [7].

Seminal research conducted by Epstein and colleagues in the early 1980s established the key role that parents play in family-based pediatric weight management interventions [8, 9]. Since these landmark studies, researchers have targeted parents as the principal agents of change in childhood overweight/obesity programs [10–13]. For example, a longitudinal study by Golan and Crow (2004) compared a child-only childhood obesity intervention to a parent-only intervention and found that weight reductions at a 7-year follow-up were greater in children from the parent-only treatment group [14]. Similarly, in 2006, Golan and colleagues compared the effectiveness of obesity interventions targeting parents exclusively versus those that included both parent and child, and found that the parent-only intervention was superior with regard to reductions in adiposity among children [15].

In addition to these early studies, numerous controlled trials have confirmed the beneficial effects of parent-focused interventions in pediatric weight management [16]. In fact, recent reviews of the literature support the conclusion that parent-only childhood obesity interventions are at least as effective as [17, 18], and potentially more effective than, parent-child and child-only approaches [19]. Furthermore, some reviews have found that parent-only interventions were more cost-effective and less resource-intensive than family-focused interventions [16, 20].

While the literature supports the efficacy of parent-focused interventions targeting childhood overweight and obesity [10–20], published papers in this area contain limited information about the key items necessary to effectively translate research into community settings [21]. The RE-AIM framework addresses such factors, vital to program generalizability and dissemination, through five evaluative dimensions–*reach*, *effectiveness*, *adoption*, *implementation*, and *maintenance* [22, 23]. RE-AIM provides a systematic means of evaluating an intervention’s potential for public health impact and widespread application, placing emphasis on both internal and external validity [22, 23].

In 2015, Jang, Chao, and Whittemore conducted a systematic review, using the RE-AIM framework, of childhood obesity treatment interventions targeting parents as agents of change [24]. Results showed that all seven of the randomized controlled trials included in the review lacked full reporting of the RE-AIM components [24]. The overall proportion of studies reporting on each dimension ranged from 23.8% (*maintenance*) to 78.6% (*reach*). Reporting on items within the *effectiveness* (60.7%), *adoption* (47.6%), and *implementation* (47.6%) dimensions was moderate amongst the studies included in the review [24]. The least reported RE-AIM items across studies were *adoption* rate (0.0%), methods of identifying the target delivery agent (0.0%), quality-of-life measures (14.3%), and estimates of costs and sustainability (0.0%).

In 2008, members of our research team developed, designed, implemented, and assessed a child-centered, community-based pilot intervention targeting childhood obesity (ISRCTN #13143236 [25]). The *Children’s Health and Activity Modification Program* (i.e., “C.H.A.M.P.”) was a 4-week family-based intervention delivered to children in a unique camp-based format. Using RE-AIM metrics, C.H.A.M.P. was found to be a potentially effective and feasible community-based childhood obesity treatment program [25]. Participation in the program was associated with several positive outcomes for children including improvements in body composition, health-related quality of life (HRQoL), and physical activity self-efficacy [25, 26]. Furthermore, focus group data revealed that parents [27] and children [28] had positive perceptions of the program. Some parents noted that future programs should include supplementary education and opportunities for parents, as well as greater parental accountability and involvement [27]. Interestingly, children also expressed a desire for increased support and involvement from their parents in order to initiate and maintain health behavior changes at home [28].

Based on the compelling empirical evidence in support of parent-focused childhood obesity treatment interventions [10–20], and key findings from the original C.H.A.M.P. pilot program [25–28], Burke and colleagues developed and implemented a second community-based pilot intervention entitled “C.H.A.M.P. Families.” This program was designed to target childhood overweight and obesity using a novel, parent-focused, group-based approach [29]. Given the paucity of studies in this area that have thoroughly reported on RE-AIM dimensions [24], the purpose of the present study was to: (a) apply the RE-AIM framework to evaluate the feasibility of this parent-focused childhood obesity intervention; and (b) explore the impact of the program on children’s HRQoL within the effectiveness dimension of RE-AIM.

## Methods

C.H.A.M.P. Families was a single-group, non-randomized, repeated measures feasibility study (ISRCTN #10752416). A detailed overview of the study protocol and theoretical foundation is published elsewhere [29]. For the purpose of the present study, a brief overview of the intervention and relevant measures is provided below.

### Participant eligibility

Parents and caregivers in London, Ontario (city population in 2017, when the study took place, was ~383,822 [30]), were eligible to participate in C.H.A.M.P. Families if: (a) they had a child between the ages of 6 and 14 years with a body mass index (BMI) ≥ 85th percentile for age and sex [31]; (b) at least one parent/caregiver agreed to take part in the study; and (c) both the child and parent(s) could speak, read, and understand English.

### Sample size

Given this was a feasibility study focused predominantly on the research and intervention process [32], a formal sample size calculation was not considered necessary [33]. Recognizing that sample sizes in such studies should be sufficient to gather the data needed to address feasibility-related questions and outcomes [34, 35], the sample size in the present study (outlined below) was deemed sufficient.

### Procedure

C.H.A.M.P. Families consisted of: (a) eight 90-min group-based (parent-only) education sessions delivered over the course of 13 weeks; (b) eight home-based (family-centered) activities; and (c) two group-based follow-up (“booster”) sessions for parents and children. Parent education sessions covered a range of topics related to child and family health (e.g., healthy eating, physical activity, screen time, family dynamics and communication, mental health) and were delivered by a number of experts and health professionals. As outlined in detail by Reilly and colleagues (2018), parent sessions were developed using evidence-based strategies grounded in social cognitive theory [36–39], motivational interviewing [40, 41], and group dynamics [42–44]. The C.H.A.M.P. Families program was free of charge, and on-site childing-minding activities were provided for all children (including siblings) during each parent session. The two family-focused booster sessions (for parents, children, and additional family members) were held 3- and 6-months post-intervention, and were designed to reinforce concepts delivered throughout the formal intervention in a fun, family-friendly atmosphere. Researchers also offered follow-up support via email and telephone up to 6-months post-intervention, after which formal contact with participants ceased. Ethical approval for all study procedures was obtained from the Health Sciences Research Ethics Board at Western University (project ID #108826). Written informed consent and assent were obtained from parents and children, respectively, prior to program involvement.

### Data collection

Parents and children completed several research assessments at four time points: baseline (i.e., ≤ 4-week pre-intervention); mid-intervention (i.e., week 6); post-intervention (i.e., week 13); and at a 6-month follow-up. For the purpose of the present study, only measures pertinent to the RE-AIM dimensions and HRQoL are presented and discussed in this section. See Table 1 for RE-AIM dimension definitions, measures, and data sources. As noted above, a detailed description of all study outcomes and measures has been published elsewhere [29].

**Table 1:** Definitions, measures, and data sources for participant characteristics and RE-AIM dimensions

Outcome(s)	Definition	Measure(s)	Data source(s)
Demographic variables	Characteristics of sample population	Primary parent: e.g., age, sex, ethnicity, marital status, education level, household income, relationship to child	Demographic survey
		Child: e.g., age, sex, years child has lived with obesity	Demographic survey
RE-AIM dimensions [22, 23]			
Reach	The absolute number, proportion, and representativeness of individuals/centers who are willing to participate in a given initiative	Eligibility criteria	Screening form
		Eligible target population estimation (valid denominator)	Statistics Canada Census, 2016
		Number of families registered for the program	Project coordinator records
		Number of families who were eligible but did not participate	Project coordinator records
		Recruitment strategies	Research records
		Identification of facilitators and barriers to recruitment	Project coordinator records
Effectiveness	The impact of an intervention on important outcomes, including potential negative effects, quality of life, and economic outcomes	Short-term attrition rates (baseline to post-intervention)	Education session attendance records
		Reasons for attrition	Project coordinator records
		Children’s health-related quality of life (child and parent-proxy reports; baseline to post-intervention)	Pediatric Quality of Life Inventory 4.0 [45]
Adoption	The absolute number, proportion, and representativeness of settings and intervention agents who are willing to initiate a program	**Staff**	
		Number of intervention agents approached to participate	Research records
		Roles and credentials of intervention agents	Research records
		**Setting**	
		Setting criteria for implementing C.H.A.M.P. Families	Research records
		Number of settings approached to implement C.H.A.M.P. Families	Research records
Implementation	The intervention agents’ fidelity to the intervention’s protocol, including consistency of delivery as intended, time and cost of the intervention, and program adaptations	Fidelity to study protocol	Research/project coordinator records
		Intervention adaptations	Research/project coordinator records
		13-week program attendance rate	Education session attendance records
		Homework completion rate	Homework records
		Financial costs of the intervention	Research records
Maintenance	The extent to which a program or policy becomes institutionalized or part of the routine organizational practices and policies	Long-term attrition rates (post-intervention to 6-month follow-up)	Project coordinator records
		**Individual**	
		Children’s health-related quality of life (child and parent-proxy reports; post-intervention to 6-month follow-up)	Pediatric Quality of Life Inventory 4.0 [45]
		**Setting**	
		Perceptions of intervention agents regarding the program and interest in future involvement	Anecdotal reports

### Measures and data analysis

#### Reach

To determine representativeness, participant demographics were compared to census demographics^1^ for the City of London, Ontario, in 2017 [30]. Records of all program inquiries were used to determine the participation rate and most effective recruitment methods via descriptive statistics of categorical variables (i.e., frequencies and proportions).

#### Effectiveness and Individual-Level Maintenance

C.H.A.M.P. Families was delivered in a real-world community setting. Thus, we evaluated *effectiveness* (rather than *efficacy*) via an examination of changes in children’s HRQoL. Children’s HRQoL was measured using the Pediatric Quality of Life Inventory (PedsQL 4.0 [45]). This inventory (*n* = 23 items) has been found to be valid and reliable, and consists of a child self-report component as well as a proxy report completed by a parent/guardian [45]. The inventory is used to assess four dimensions of children’s quality of life (i.e., *physical*, *emotional*, *social*, and *school*), which were aggregated in accordance with the scoring manual to generate two summary scores (i.e., a Physical Health Summary Score and Psychosocial Health Summary Score) for subsequent analysis [45].

Given the nature of our study (and small sample size), a quasi-experimental single-subject design with intersubject replication was used to examine the HRQoL data, wherein each participant served as their own control [46, 47]. Potential relationships between the intervention and HRQoL were identified through the intensive and prospective study of individuals over time (i.e., repeated measures [46, 47]). Single-subject data were examined through visual analysis of both *level* (i.e., change scores from baseline to post-intervention and post-intervention to 6-month follow-up) and *trend* (i.e., slope from baseline to 6-month follow-up [46, 47]). An increase in score (*level*) or slope (*trend*) represents a positive outcome and indicates an improvement in children’s HRQoL throughout the study period. Minimal clinically important differences^2^, considered to represent the smallest difference in a score that is perceived to be beneficial for the individual [49], are reported in the results section.

#### Adoption

Data and detailed descriptions (i.e., roles, credentials, demographic information, and/or representativeness where applicable) of delivery settings and intervention agents (i.e., researchers, setting staff, guest speakers) involved in the implementation of the program were recorded by the research team and analyzed via descriptive statistics of categorical variables (i.e., frequencies and proportions).

#### Implementation

Records detailing anticipated and actual planned program activities/components were kept by the research team to determine whether the intervention was delivered as intended. Take-home (i.e., family homework) activities were also assessed for completion and recorded as a measure of participants’ use of implementation strategies. Sign-in sheets supplied at the parent education and booster sessions were used to track participant attendance and retention. While most study-related data were collected from the primary parent only, researchers recorded the attendance of all family members and invited them to join the education sessions, booster sessions, and post-intervention focus groups. Lastly, costs associated with the development, implementation, and delivery of the program were documented in detail (including in-kind contributions).

#### Maintenance

*Maintenance* is assessed at both individual and setting levels [22, 23]. As noted above, *individual-level maintenance* was considered and assessed as part of program *effectiveness*. *Setting-level maintenance* was assessed via the use of anecdotal reports of interest from individuals who were approached and took part in the C.H.A.M.P. Families intervention at a setting/delivery level (i.e., intervention agents).

## Results

### Participants

Twenty-three parents representing 25 children inquired about the program during the 4-month recruitment period. Of these, 17 parents (74.0%) were assessed for eligibility. Of the 15 families who were deemed eligible, 11 families (73.3%) agreed to participate in the program. In total, 16 parents representing 11 children (six dyads and five triads) enrolled. At baseline, the self-reported age of the primary parents (*n* = 11) and children (*n* = 11) ranged from 30 to 52 years (*M*_Age_ = 42.0 years, *SD* = 6.4), and 6 to 14 years (*M*_Age_ = 9.5 years, *SD* = 2.0), respectively. All children had a *BMI* ≥ 95th percentile for age and sex (BMI-*z* scores ranged from 1.74–2.75; *M*_BMI-*z*_ = 2.20, *SD* = 0.3); see Table 2 for an overview of demographic information for parents and children.

**Table 2:** Baseline demographic information for parents* and children involved in C.H.A.M.P. Families

Demographic variable	*n* (%)
**Parent** (*n* = 11)	
Age (mean ± *SD*)	42 (6.4)
Sex	
Female	10 (90)
Male	1 (10)
Ethnicity	
Caucasian	8 (73)
Arab	2 (18)
Egyptian	1 (9)
Marital status	
Married	8 (73)
Common law	1 (9)
Separated	1 (9)
Single	1 (9)
Educational attainment	
University degree	7 (64)
College diploma	2 (18)
Trades certificate	1 (9)
High school diploma	1 (9)
Employment	
Full-time	6 (55)
Part-time	3 (27)
Unemployed	2 (18)
Household income	
≥ $100,000	4 (36)
$70,000–$79,999	2 (18)
$60,000–$69,999	2 (18)
$50,000–$59,999	1 (9)
$30,000–$39,999	1 (9)
$20,000–$29,999	1 (9)
**Child** (*n* = 11)	
Age (mean ± *SD*)	9.5 (2.0)
Sex	
Female	7 (64)
Male	4 (36)
BMI-*z* (mean ± *SD*)	2.2 (0.3)
Weight issue (mean ± *SD*)	~4.9 yrs. (2.5)

Note: *Demographic information was only collected from the self-identified primary parent in each family

**Fig. 1:**
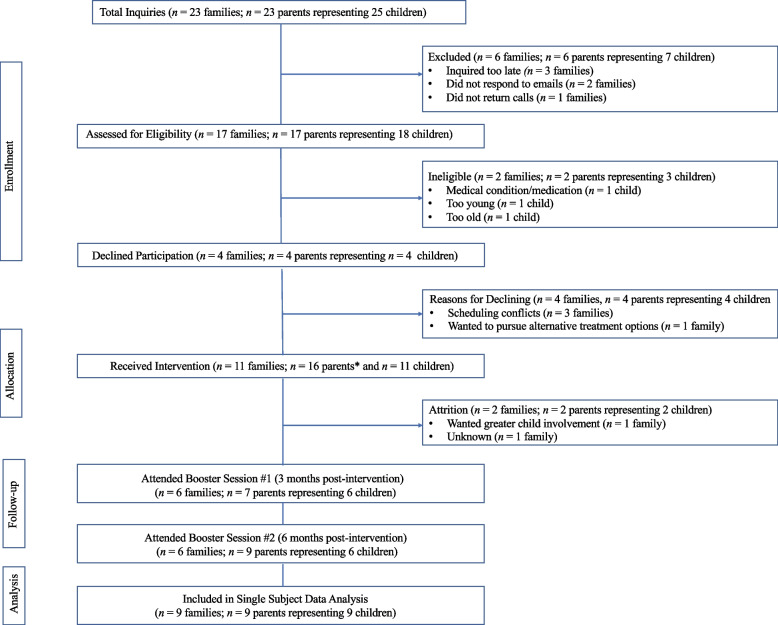
Consolidated Standards of Reporting Trials (CONSORT) diagram for intervention recruitment and attrition Note: *Of the 11 self-identified primary parents, 5 attended the educational and/or booster sessions with a secondary parents/caregiver (i.e., parent or stepparent)

Figure 1 provides an overview of the participation and attrition rates for C.H.A.M.P. Families, including reasons for ineligibility and dropout. One parent-child dyad withdrew from the program at week 4, and another was lost to follow-up at week 6 of the 13-week intervention, resulting in a total of nine families who completed the formal intervention.

### Reach

Data regarding the number of potentially eligible families within the defined target population were not available for the area in which the program was offered. However, as of 2016, there were 41,585 children aged 5–14 living in London, Ontario [30]; of which, approximately 30% were considered to have been living with overweight/obesity [50]. Taking into account these data [30, 50], and recognizing the uncertainty as to whether these individuals would have been eligible for the program, it is estimated that C.H.A.M.P. Families reached approximately 0.09% of families with children between the ages of 5 and 14 living with overweight/obesity in the London, Ontario, community.

Based on Statistics Canada data for the city in which the program took place [30], families appeared to be similar to the population from which they were drawn in terms of ethnicity (72.7% and 80.0% identified as Caucasian in C.H.A.M.P. Families and London, respectively), income (median household income was $70,000–$79,999 and $62,011 CAD, respectively), and employment status (81.8% and 75.6% employed, respectively). With regard to self-reported sex, only five parents (31.3%) and four children (44.4%) identified as male. While females marginally outnumber males in London, Ontario (i.e., 52% female and 48% male [30]), male parents and male children were underrepresented in the current study.

As for participant recruitment, the strategies used to recruit eligible families, from most to least effective, were radio advertisements (*n* = 4 families, 33.3%), word of mouth (*n* = 3 families, 25.0%), newspaper advertisements (*n* = 2 families, 16.7%), social media (*n* = 2 families, 16.7%), and physician referrals (*n* = 1 family, 8.3%). It should be noted that one family indicated that they heard about the program from two sources (i.e., radio and Internet/social media).

### Effectiveness and Individual-Level Maintenance

As noted above, a total of nine parent-child dyads completed the full intervention; thus, only these families were included in the HRQoL analysis. There were no cases of missing data across all assessment points for these nine families.

### Child-reported HRQoL

With regard to *level*, seven of nine children (77.8%) reported an increase in physical HRQoL scores across the 13-week intervention. From post-intervention to the 6-month follow-up, only one child (11.1%) reported an increase in physical HRQoL, although five (55.6%) children maintained a minimal clinically important difference (i.e., a change of 6.66) across the entire study period. Similarly, six of nine children (66.7%) reported an increase in psychosocial HRQoL scores from baseline to post-intervention. From post-intervention to the 6-month follow-up, four of nine children (44.4%) reported an increase in psychosocial HRQoL, and six children (66.7%) maintained a minimal clinically important difference (i.e., a change of 5.30). Change scores from baseline to post-intervention and post-intervention to 6-month follow-up (for child and parent-proxy reports) are displayed in Tables 3 and 4.

**Table 3:** Change scores for child- and parent-reported *physical health-related quality of life* (HRQoL)* for children across study time points (summary change scores)

	Child-reported physical HRQoL		Parent-proxy ratings of children's physical HRQoL	
Participant ID	Baseline–Post-intervention	Post-intervention–Follow-up	Baseline–Post-intervention	Post-intervention–Follow-up
1	+15.62	−9.37	+12.50	−15.62
2	+28.13	−6.25	+18.75	0.00
3	+21.87	−12.50	0.00	−6.25
4	+3.12	+9.38	+12.50	−6.25
5	+3.13	0.00	+3.12	+9.38
6	+28.12	−6.25	+28.12	+3.13
7	0.00	−3.12	−12.52	+6.37
8	−6.25	−34.37	−40.63	+40.63
9	+40.62	0.00	−12.05	+9.37
Mean (*SD*)	14.93 (15.84)	−6.94 (12.08)	1.09 (20.64)	4.53 (15.87)

Note: *Changes in HRQoL were determined using the PedsQL4.0 assessment tool [45]

**Table 4:** Change scores for child- and parent-reported *psychosocial health-related quality of life* (HRQoL)* for children across study time points (summary change scores)

	Child-reported psychosocial HRQoL		Parent-proxy ratings of children's psychosocial HRQoL	
Participant ID	Baseline–Post-intervention	Post-intervention–Follow-up	Baseline–Post-intervention	Post-intervention–Follow-up
1	+18.33	+6.67	+18.34	−31.67
2	+28.34	−3.34	+23.33	−6.66
3	+10.00	+6.66	+8.30	−8.33
4	0.00	0.00	+3.33	+6.67
5	+16.67	−2.03	+16.67	−13.33
6	+6.67	0.00	+11.67	−12.74
7	−10.00	+5.00	+16.67	−23.33
8	+16.66	−13.33	+6.66	−1.66
9	−3.34	+20.00	0.00	+1.67
Mean (*SD*)	9.26 (12.14)	2.18 (9.11)	11.66 (7.69)	−9.93 (12.06)

Note: *Changes in HRQoL were determined using the PedsQL4.0 assessment tool [45]

**Fig. 2:**
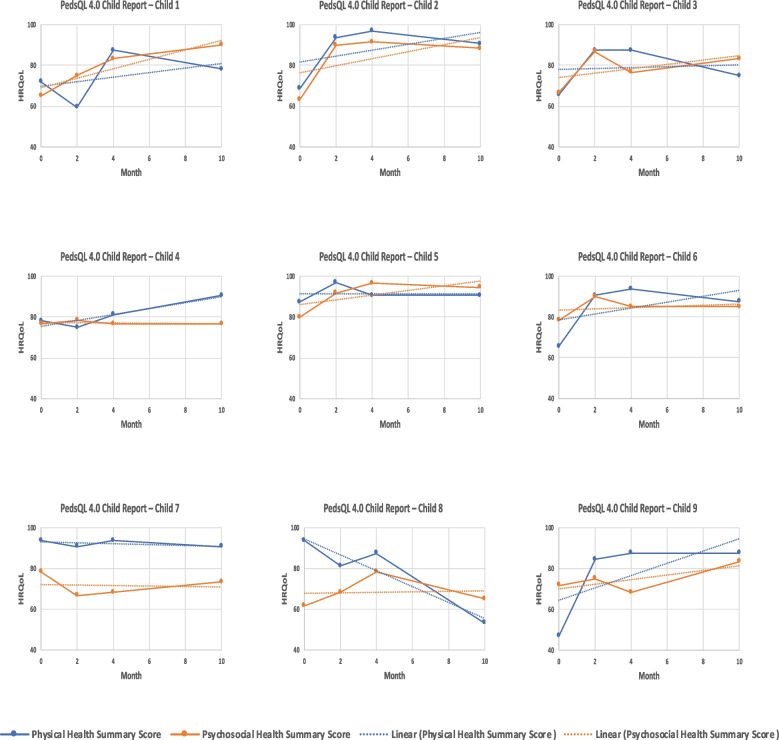
Graphed data and trends for child-reported health-related quality of life (HRQoL)

**Fig. 3:**
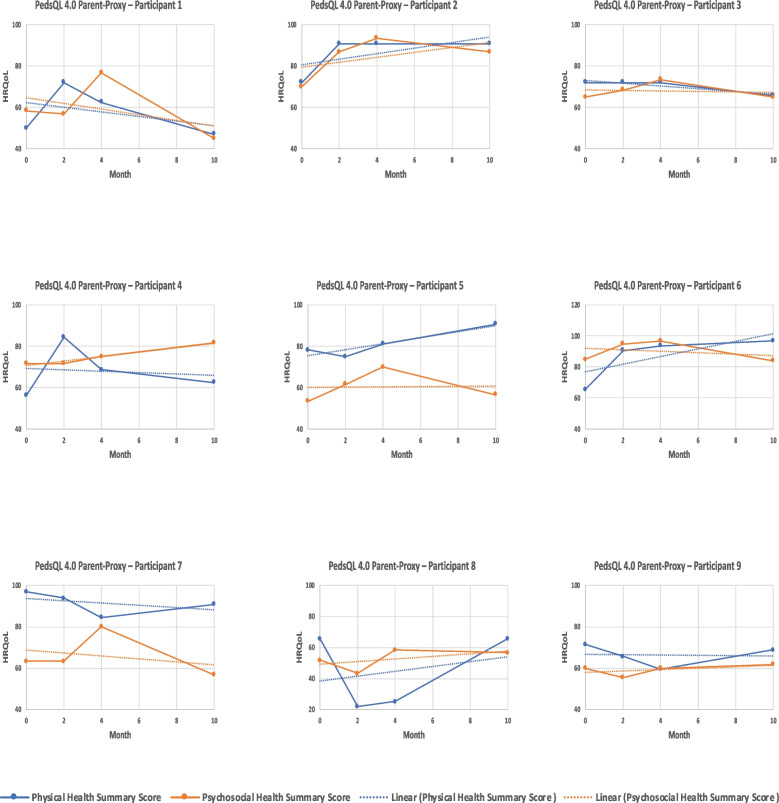
Graphed data and trends for parent-reported children’s health-related quality of life (HRQoL)

As for *trend*, six of nine children (66.7%) reported positive changes in physical HRQoL across the entire study period. Positive trends in child-reported psychosocial HRQoL were observed for seven of nine children (77.8%) from baseline to 6-month follow-up. Trends of individual child-reported summary scores are displayed in Tables 5 and 6. Corresponding graphed data can be found in Figs. 2 and 3 in the Appendix.

**Table 5:** Trends from baseline to 6-month follow-up for child- and parent-reported *physical health-related quality of life* (HRQoL)* for children (summary scores)

	Trends from baseline to 6-month follow-up	
Participant ID	Child-reported physical HRQoL	Parent-proxy ratings of children's physical HRQoL
1	+1.12	−1.12
2	+1.45	+1.34
3	+0.22	−0.67
4	+1.45	−0.34
5	0.00	+1.45
6	+1.45	+2.46
7	−0.22	−0.55
8	−3.91	+1.56
9	+3.01	−0.08
Mean (*SD*)	0.51 (1.93)	0.45 (1.26)

Note: *Changes in HRQoL were determined using the PedsQL4.0 assessment tool [45]

**Table 6:** Trends from baseline to 6-month follow-up for child- and parent-reported *psychosocial health-related quality of life* (HRQoL)* for children (summary scores)

	Trends from baseline to 6-month follow-up	
Participant ID	Child-reported psychosocial HRQoL	Parent-proxy ratings of children's psychosocial HRQoL
1	+2.32	−1.37
2	+1.73	+1.19
3	+1.07	−0.12
4	−0.06	+1.07
5	+1.15	+0.06
6	+0.30	−0.47
7	−0.12	+0.71
8	+0.12	+0.83
9	+1.13	+0.34
Mean (*SD*)	0.85 (0.85)	0.25 (0.82)

Note: *Changes in HRQoL were determined using the PedsQL4.0 assessment tool [45]

### Parent-reported HRQoL

Insofar as *level* is concerned, five of nine parents (55.6%) reported an increase in their child’s physical HRQoL during the formal 13-week intervention. From post-intervention to the 6-month follow-up, five of nine parents (55.6%) reported an increase in their child’s physical HRQoL, three of which were considered minimal clinically important differences (i.e., a change of 6.92). Moreover, eight of nine parents (88.9%) reported an increase in child psychosocial HRQoL from baseline to post-intervention. At 6-month follow-up, two parents (22.2%) reported an increase in psychosocial HRQoL from the post-intervention scores, although only one was considered a minimal clinically important difference (i.e., a change of 5.49). Change scores for parent-reported child HRQoL from baseline to post-intervention and post-intervention to 6-month follow-up are displayed in Tables 3 and 4.

Pertaining to the observed *trend* in parent-reported child physical HRQoL, four of nine parents (44.4%) reported positive changes across the full study period (i.e., from baseline to 6-month follow-up). Increases in *trend* scores for parent-reported child psychosocial HRQoL were reported by six of nine parents (66.7%) across the study period. Trends of individual parent-proxy summary scores are displayed in Tables 5 and 6.

#### Adoption

One primary community-based delivery setting (the YMCA) was involved in the implementation of C.H.A.M.P. Families. A central YMCA location was selected to host the 13-week intervention based on its suitability and family-friendly environment, as well as an ongoing working relationship and prior involvement in the original C.H.A.M.P. program [25]. A boardroom was provided, at no cost, in which the eight group-based parent education sessions were delivered. Two additional rooms were also provided to host the end-of-program focus groups.

All of the intervention agents (i.e., program staff/personnel; *n* = 29) who were approached to participate in the design and delivery of the 13-week intervention agreed to do so. Seven of these individuals (24.1%) were co-investigators/researchers who contributed to the design and development of C.H.A.M.P. Families, 12 (41.4%) were considered content experts (i.e., researchers, health professionals, and/or other experts in the area[s] of interest) responsible for delivering the parent-focused education sessions, and six (20.7%) provided support services (i.e., reception, child-minding).

Three months after the conclusion of the formal intervention period, the first family-focused booster session was held at a local not-for-profit organization focused on enhancing and promoting food education and literacy among children and families. Delivery agents included three professional chefs and three volunteers (representing 20.7% of 29 intervention agents) from this organization to facilitate the 2-h family event. At 6-months post-intervention, the second booster session was held at a local obstacle course center focused on physical activity and physical literacy. Two staff members from this organization (6.9% of 29 intervention agents) facilitated this booster session for parents and children.

#### Implementation

All (100%) of the parent-focused education sessions (*n* = 8), home-based goal-setting activities (*n* = 8), and follow-up booster sessions (*n* = 2) were implemented as planned. Additional resources, developed in response to parent requests (e.g., C.H.A.M.P. Families Community Resources Handbook), were provided to participants throughout the intervention. As a result, more resources were distributed than originally planned. The completion rate of the eight assigned home-based goal setting worksheets was, on average, one (12.5% of assigned worksheets) per family.

As noted above, attendance records and homework completion logs were reviewed to assess participants’ use of intervention and implementation strategies (i.e., *individual-level implementation*). The average attendance rate for the parent-only educational sessions was 78.9%. Program attendance was found to be higher among participants who had a secondary caregiver attend the sessions with them (*M*_Attendance_ = 97.5% or 7.8/8.0 sessions) than for participants who did not have a secondary caregiver present at sessions (*M*_Attendance_ = 62.5% or 5.0/8.0 sessions). Lastly, average parental attendance for the family-focused booster sessions was 46.7% for the first session and 60.0% for the second session.

The C.H.A.M.P. Families budget was divided into four general cost categories: personnel ($19,150.00 CAD); research and recruitment ($7,550.00 CAD); equipment and supplies ($3,900.00 CAD); and knowledge dissemination ($3,800.00 CAD). In total, the funds required to design and implement C.H.A.M.P. Families, excluding external researcher and graduate student funding received from grants and awards, were approximately $34,400.00 CAD.

#### Setting-Level Maintenance

Because C.H.A.M.P. Families was a feasibility study focused primarily on the implementation of a 13-week community-based intervention [32], long-term maintenance was not assessed. However, anecdotal reports from individuals representing organizations who took part in (at a setting/delivery level) the C.H.A.M.P. Families intervention expressed a keen interest in participating in future projects.

## Discussion

The purpose of this study was to use the RE-AIM framework [22, 23] to determine the feasibility of C.H.A.M.P. Families, a parent-focused intervention targeting childhood obesity. While the program had limited *reach* (~0.09% of eligible families living in the city in which the intervention took place), the participation rate of families who inquired about the program and were eligible was high. Parents who enrolled in C.H.A.M.P. Families (*n* = 16) were predominantly female, Caucasian, married, employed, and had some form of postsecondary education. Bearing in mind the small sample size, it was found that participant ethnicity, employment status, and median household income were generally representative of the broader community [30]. C.H.A.M.P. Families was designed to address a number of challenges to participation and retention that have been noted in the childhood obesity literature [51, 52]; the program was offered to participants at no cost, had few exclusion criteria, utilized low intensity and timely implementation strategies (i.e., 12 h over 13 weeks), and included complimentary parking and child minding for all children (including siblings).

While data for numerous health-related outcomes (e.g., standardized body mass index [BMI-*z*], physical activity levels and sedentary time) were collected as part of the larger study [29], children’s HRQoL was used as the indicator of *effectiveness* (i.e., from baseline to post-intervention) and *individual-level maintenance* (i.e., from post-intervention to the 6-month follow-up) in this study for several reasons. First, HRQoL has been recognized as an important consideration in the childhood obesity treatment literature [53, 54], as well as within the context of the RE-AIM framework [22, 23]. Second, while C.H.A.M.P. Families was designed specifically for parents of children with obesity (and children’s BMI-*z* was indeed a primary outcome [29]), the intervention was created, as outlined in the program philosophy, “…to improve family health behaviors and communication by enhancing the knowledge and confidence of parents in a group-based environment that is safe, supportive, inclusive, and positive.” Therefore, rather than focusing on weight loss, parents and families were supported in an effort to make lifelong healthy choices and behavior changes that were sustainable and realistic. Third, and in line with the previous reason for utilizing HRQoL as an important measure of effectiveness, it has been suggested that focusing primarily on anthropometric outcomes in childhood obesity research may be problematic and even detrimental to children’s health and well-being [55]. Instead, encouraging healthy behaviors alongside positive communication may result in better health outcomes [56].

The majority of children in the present study reported increases in both physical and psychosocial HRQoL summary scores over the 13-week program. As for *individual-level maintenance*, while the majority of children reported reduced scores in both physical and psychosocial health at the 6-month follow-up, minimal clinically important differences remained for some children’s physical and psychosocial HRQoL summary scores (*n* = 5 and 6 out of 11, respectively). The fact that C.H.A.M.P. Families may have had a lasting (i.e., ≥ 6 months) minimal clinically important impact on psychosocial HRQoL for some children is noteworthy, as Tsiros and colleagues (2009) have suggested that psychosocial functioning among children may be more resistant to change than physical functioning [54]. These findings are also in line with other pediatric obesity studies showing that HRQoL scores among children with obesity tend to increase during behavioral-based treatments, and such improvements are generally maintained (although often lower than post-intervention scores) up to 1-year follow-up [25, 54]. Moreover, the apparent positive impact of the C.H.A.M.P. Families program on child-reported HRQoL is particularly important given the intervention was delivered to parents only, without child involvement. Whether this reflects the effectiveness of the educational intervention, the motivation of parents to improve their child’s health and HRQoL, or a combination of these and other factors, requires further investigation.

Generally speaking, parents reported lower child HRQoL scores than children, with fewer reported clinically important improvements in both physical and psychosocial HRQoL summary scores from baseline to the 6-month follow-up. Differences in parent and child HRQoL scores are not uncommon in the literature [57] and are important to acknowledge as it has been suggested that parents’ perceptions of their child’s HRQoL often influence treatment-seeking behaviors [58].

With regard to *adoption* and *setting-level maintenance*, 100% of the individuals and community organizations approached to participate in the design and/or delivery of C.H.A.M.P. Families agreed to take part. Designing interventions that include a variety of community-based organizations and account for stakeholder priorities can improve community partner involvement and program sustainability [59, 60]. As such, all intervention agents who served as members of the core research team or were approached to deliver aspects of the program were involved to some extent in the planning, development, and/or delivery of the intervention. As C.H.A.M.P. Families was delivered predominantly by highly specialized health professionals and support personnel, an important point of consideration is that while most of these sessions were provided free of charge, sustained adoption/integration in community-based settings—if delivered in the same format by the same individuals—may pose a challenge.

A considerable strength of C.H.A.M.P. Families was that numerous community partnerships, vital to the success of the program, were initiated and maintained. For example, the local YMCA was fundamental in providing a safe, family-friendly venue for program delivery and recreation-based child minding. Furthermore, the successful *adoption* of the program within the local community, and its potential for *setting-level* sustainability, is reflected in continued community-based support of C.H.A.M.P.-related projects aimed at the treatment of childhood obesity [25].

In terms of *implementation*, although the percentage of planned activities implemented was high across the 13-week intervention, completion of the eight home-based activities was low. Focus groups conducted with parents during the final C.H.A.M.P. Families session revealed that time constraints were perceived as a barrier to health behavior change among participants [61]. Thus, it is possible that the home-based activities were viewed as an additional burden beyond the time commitment already required for the intervention.

With regard to program adherence, overall parental attendance was high, with waning retention across the two booster sessions. The high level of adherence to the formal intervention is noteworthy as adherence and attrition issues are commonly cited as challenges in childhood obesity intervention research, particularly for programs that target parents [16, 18, 24]. The theoretical foundation of C.H.A.M.P. Families, including the use of evidence-based group dynamics strategies and motivational interviewing techniques [29], coupled with regular participant contact, may have played a role in promoting program adherence.

Lastly, another aspect of *implementation* that merits discussion is the cost associated with developing and implementing a community-based program such as C.H.A.M.P. Families. Reporting the costs of an intervention is considered important when attempting to enhance program translation [62]. Unfortunately, there is a lack of reported implementation costs in the parent-focused childhood overweight and obesity literature [24]. Our results show that it was possible to implement a parent-focused, community-based childhood obesity intervention at a relatively low cost, without extensive external funding, which aligns with evidence suggesting that parent-only childhood obesity interventions are typically less expensive and require fewer resources than those that involve children directly [16, 20]. Such findings certainly lend support to the idea that a program similar to C.H.A.M.P. Families may be sustainable, translatable, and cost-effective to implement in a community setting.

### Limitations and future directions

Notwithstanding the apparent positive impact and potential of this parent-focused program, several limitations should be noted. First, despite extensive recruitment efforts, the sample size of C.H.A.M.P. Families was small (11 children and 16 parents/caregivers at baseline). Although participants appeared to be representative of the population in which the intervention took place, the low sample size and resultant single-subject analyses conducted preclude any possibility for generalization of findings. As noted by Reilly and colleagues (2018), future attempts to enhance recruitment for parent-focused, community-based pediatric obesity interventions could include a longer recruitment period, enhanced program messaging and marketing, greater child involvement, and additional family-based activities [29].

The single community-based setting used for C.H.A.M.P. Families, as well as high specificity of trained staff involved in the delivery of the program, could also limit generalizability. As the formal 13-week intervention was implemented at a single site, reporting of setting comparison information (e.g., reasons for participation vs. non-participation) was not possible. Moreover, translation to other locations, particularly rural and remote settings, may pose additional challenges. Families living in such areas may have reduced access to health-related services [63] and face unique geographical burdens including transportation issues, extreme weather, and food insecurity [64].

Given the apparent feasibility and preliminary effectiveness of C.H.A.M.P. Families, next steps include the design, implementation, and evaluation of a randomized controlled trial (RCT) to test the efficacy and cost-effectiveness of the intervention [65]. While C.H.A.M.P. Families was strategically designed as community-based, lifestyle intervention for parents of children with obesity, Reilly and colleagues (2019) noted that some parents who took part in the intervention felt that their children would have benefited from increased participation in the program [61]. Given the ample literature supporting family-based interventions in which parents are the primary agents of change [10–20], establishing a balance between program design and effectiveness/efficacy and participant preferences is imperative. Ensuring that parents and families are ready to commit to a parent-focused intervention will be an important aspect of the successful implementation and sustainability of future pediatric obesity initiatives. Additionally, to maximize translation and scalability of the C.H.A.M.P. Families program, it is important to consider design, recruitment, and implementation strategies to better serve and target more diverse geographic areas and populations. Lastly, iterative application of the RE-AIM framework in both planning and evaluation can inform meaningful adaptations, enhancing the reach, effectiveness, and potential adoption of future interventions [23, 66, 67].

## Conclusion

In short, C.H.A.M.P. Families holds promise as a parent-focused treatment intervention for children with obesity. The current paper includes a comprehensive examination of, and detailed reporting on, key elements within each dimension of the RE-AIM framework. Together, these findings provide important and pragmatic information which can be used to inform the development and implementation of community-based pediatric obesity programs. It is also expected that the findings herein will inform the design and delivery of a future RCT conducted with a larger, more diverse group of children and families. Moving forward, researchers should consider the use of RE-AIM in both the planning and evaluation stages of interventions targeting childhood obesity.

## Data Availability

The datasets during and/or analyzed during the current study are available from the corresponding author on reasonable request.

## Appendix

Figures 2 and 3

## Abbreviations

BMI: Body mass index
BMI-*z*: Standardized body mass index
C.H.A.M.P.: Children’s Health and Activity Modification Program
HRQoL: Health-related quality of life
PedsQL 4.0: Pediatric Quality of Life Inventory 4.0
RE-AIM: Reach, effectiveness, adoption, implementation, and maintenance

## Footnotes

1 The C.H.A.M.P. Families intervention was implemented in the City of London, Ontario, in 2017. Census data were drawn from the Statistics Canada 2016 Census for the City of London [30]. Given the location in which the intervention took place (as well as where participants were recruited), data from the surrounding metropolitan areas were not included in analyses.

2 A change in the Physical Health Summary score of 6.66 (child) and 6.92 (parent proxy), and in the Psychosocial Health Summary Score of 5.30 (child) and 5.49 (parent proxy), constitutes a minimal clinically important difference, as reported in the PedsQL 4.0 scoring manual [45, 48].
